# Safety and efficacy of combined trastuzumab-deruxtecan and concurrent radiation therapy in breast cancer. The TENDANCE multicentric French study^☆^

**DOI:** 10.1016/j.breast.2025.104421

**Published:** 2025-02-13

**Authors:** K. Debbi, M.A. Benderra, J. Medioni, C. Durdux, N.H. To, C. Boukhobza, N. Grellier, A. Benmaziane, L. Monnier, J. Gligorov, E. Assaf, Y. Belkacemi

**Affiliations:** a Department of Radiation Oncology and Henri Mondor Breast Center. Henri Mondor University Hospital, APHP, UPEC, Créteil, France; b Institut Mondor de Recherche Biomédicale (IMRB), INSERM U955, i-Biot, UPEC, Créteil, France; c Medical Oncology Department, Institut Universitaire de Cancérologie Assistance Publique-Hôpitaux de Paris-Sorbonne Université, Paris, France; d Centre of Early Clinical Trials in Cancer, Hôpital Européen Georges-Pompidou, Université Paris Cité, Paris, France; e Department of Radiation Oncology, European Hospital Georges Pompidou, University of Paris Descartes, Paris, France; f Medical Oncology Department, Hôpital Foch, Paris, France; g Radiation Oncology Department, DMU Orphé, Hôpital Tenon, AP-HP.Sorbonne Université, France; h Department of Medical Oncology, The Henri Mondor University Hospital, Creteil, France

**Keywords:** Trastuzumab deruxtecan, Radiotherapy, Combined therapy, Safety, Efficacy

## Abstract

**Purpose:**

Trastuzumab Deruxtecan (T DXD), a new antibody drug conjugate is a new treatment option for 2nd line metastatic breast cancer (MBC) for HER2 + or HER2 low tumors. Palliative or ablative radiotherapy (RT) may be required in patients who are being treated with T-DXd. However there is a lack of evidence regarding the safety profile of combining T-DXd with RT. TENDANCE study aimed to evaluate safety and efficacy of combined T-DXd and RT.

**Materials and methods:**

This retrospective multicenter study included 54 patients treated concurrently with T-DXd and RT for HER2+ and HER2 low MBC between February 2021 and December 2023. All data were collected from a web-questionnaire, centralized after medical records and validation of the protocol by the local ethical committee. Primary endpoint was the safety of combined therapy.

**Results:**

Median age was 60 years. Patients who received T-DXD were further categorized into HER2+ (40.7 %), HER2 low/hormonal receptors HR+ (40.8 %) or HER2 low/HR- (18.5 %). In the HER2+ patients, T-DXd was administered as 2nd (18.2 %) or 3rd (31.8 %) or 4th (50 %) line therapy. RT was delivered using palliative (72.2 %) or ablative doses (27.8 %). Indications consisted mostly of palliative bone irradiation (46.3 %) and stereotactic radiosurgery (SRS) (25.9 %). With the median follow-up of 9 months, 22.2 % of patients had a complete response and 77.8 % had either a partial response or stable disease. Grade 1 or 2 asthenia was observed in 51.8 % of patients, while only 16.6 % experienced other grade 1 or 2 adverse effects. There was no T-DXd therapy discontinuation related to RT.

**Conclusion:**

To our knowledge, TENDANCE is the largest study evaluating concurrent T-DXd and RT. This preliminary report suggests the feasibility of the combination of RT and T-DXd with manageable toxicity rate. Longer follow-up and further prospective studies are required to confirm these results.

## Highlights

• TENDANCE cohort is to date the largest study evaluating safety and efficacy of concurrent RT in patients treated with T-DXd.
• T-DXd and RT showed minimal and manageable grade 1–2 toxicity, with optimal clinical benefit observed.
• Our results seem encouraging for clinical practice, underscoring the feasibility and safety of this combined approach.

## 1 Introduction

Breast cancer is the most frequently diagnosed and the leading cause of cancer-related deaths among women worldwide. By 2040, the incidence of new breast cancer cases across all six continents is projected to increase by 49 % from 2020 levels, with death incidence expected to rise by 62 % [1].

Breast cancer is categorized into three major subtypes based on molecular markers for estrogen or progesterone receptors and human epidermal growth factor receptor 2 (HER2): hormone receptor (HR)-positive/HER2-negative (70 % of patients), HER2-positive (15–20 %), and triple-negative (tumors lacking all three standard molecular markers; 15 %) [2].

The standard treatment for newly diagnosed HER2-positive (HER2+) metastatic breast cancer is a combination of pertuzumab and trastuzumab (anti-HER2 antibodies) with taxanes [[3], [4], [5]]. For patients whose disease progresses after this treatment, the standard second-line treatment is trastuzumab deruxtecan (T-DXd). The DESTINY Breast 03 trial indicated that treatment with T-DXd resulted in a median progression-free survival of 28.8 months compared with 6.8 months with trastuzumab emtansine [6].

Approximately 60 % of HER2-negative metastatic breast cancers express low levels of HER2, defined as an immunohistochemical (IHC) score of 1+ or an IHC score of 2+ with negative results on in situ hybridization (ISH) [7,8].

T-DXd is an antibody-drug conjugate (ADC) consisting of a humanized anti-HER2 monoclonal antibody linked to a topoisomerase I inhibitor payload through a tetrapeptide-based cleavable linker [9]. T-DXd is a new ADC particularly recommended for the treatment of HER2-overexpressing (IHC 3+ or IHC 2+/ISH+) and HER2-low (IHC 2+/1+ and ISH-) metastatic breast cancer (MBC) [[10], [11], [12]]. T-DXd is typically administered as an intravenous infusion every 3 weeks.

Patients with metastatic disease may receive radiotherapy (RT) for palliative or ablative purposes combined with T-DXd.

Currently, data on the concurrent use of radiotherapy and trastuzumab deruxtecan (T-DXd) are limited. While the DESTINY-Breast 03 trial demonstrated the efficacy of T-DXd in patients with previously treated HER2-positive metastatic breast cancer, it did not specifically assess the safety or efficacy of concurrent T-DXd and radiotherapy administration [6]. This is particularly important given the significant risk of radionecrosis that has been reported when combining brain irradiation with trastuzumab emtansine [20].

Therefore, TENDANCE study aims to address this gap by providing data on the safety and efficacy of this combination therapy.

## 2 Materials/methods

Between February 2021 and December 2023, patients treated in five French centers were included in the TENDANCE study.

The inclusion criteria were to be undergoing every 3 weeks treatment with T-DXd and to have received concurrent radiation. All included patients had started T-DXd before the initiation of RT and continued it after the final RT session. This includes cases where RT was administered between two T-DXd cycles as well as those where RT and T-DXd were given concurrently. To select the patients included in our study, we retrieved from each center the list of all patients treated with T-DXd and then verified if they had received radiotherapy during this period. After identifying all patients who had received radiotherapy during their treatment with T-DXd, no patients were excluded. All data were collected from a web-questionnaire, centralized after medical records after the validation of the protocol by the local ethical committee.

The questionnaire included four sections. The first section was dedicated to the disease characteristics and T-DXd administration. The second section was focused on the RT parameters. The rest of the questionnaire was devoted to toxicity and efficacy of the combined therapy. All clinical and technical information was available on a patient management software chart. The referring doctors were responsible for completing the questionnaire for their respective patients.

Primary outcome of the study was the safety of combined T-DXd and concurrent RT. Toxicities were assessed according to the Common Terminology Criteria for Adverse Events (CTCAE) Version 5.0. All patients were evaluated during a post-radiation consultation a few days after the last RT session, then at 3 and 6 months.

The second outcome was the efficacy of this association which was categorized as complete response, partial response, stable disease, or disease progression. The assessment was radiological tool according to RECIST criteria. The assessment was based on radiological evaluation according to RECIST criteria. All patients underwent conventional imaging assessments every three months, which could include thoraco-abdominopelvic CT scans, FDG PET-CT scans, and, in cases of brain metastases, brain MRI. The choice of imaging modality depended on the disease status and the localization of metastases.

The five centers that participated in this study belong to the same institution and follow equivalent patient management methodologies. A post-radiotherapy consultation was systematically conducted at the end of treatment, during which adverse events were reported. Moreover, in the context of metastatic breast cancer management, the quarterly assessment procedures are standardized.

Patients characteristics, efficacy and toxicity results were summarized with descriptive statistics, including medians, means, and standard deviations for continuous data. Follow-up was conducted from the last day of radiotherapy until death or the last examination. All analyses were performed using R software version 3.5.1 (R project, Vienna, Austria).

## 3 Results

### 3.1 Trastuzumab deruxtecan administration

Data of 54 MBC patients treated with T-DXd and concurrent RT were collected. The median age was 60 years [39.6–85.7] and the average age was 61 years. Among the patients, 70.4 % were under 65 years old, while 24.1 % were over 70 years old. In terms disease type, 25.9 % of the patients were treated for HER2+/HR + MBC, 14.8 % for HER2+/HR- MBC, 40.7 % for HER2 low/HR + MBC and 18.5 % for HER2 low/HR- MBC.

For the twenty-two HER2+ patients, T-DXd was administered from the second to the seventh line therapy. T-DXd was administered as 2nd (18.2 %) or 3rd (31.8 %) or 4th (22.7 %), or 5th (18.2 %), or 6th (4.5 %) or 7th (4.5 %) line therapy. For the ten HER2 low/HR-patients, T-DXd was also delivered from the second to the seventh line. T-DXd was delivered as 2nd line (20 %), or 3rd line (20 %), or 4th line (40 %) or 7th line (20 %) therapy. For the twenty-two HER2 low/HR + patients, T-DXd was given as salvage treatment between the 2nd and the 11th line therapy. T-DXd was given as 2nd line (9.1 %), 3rd line (22.7 %), 4th line (18.2 %), 5th line (27.3 %), 6th line (9.1 %), or 7th line (4.5 %), 9th line (4.5 %) or 11th line (4.5 %) (Table 2).

All patients received a T-DXd dose of 5.4 mg/kg, except for 4 patients who were treated with a lower dose of 4.4 mg/kg.

The median time between the first dose of T-DXd and the first RT session was 48 days [4–498] while the average time was 86 days. The median time between the last dose of T-DXd and the first RT session was 10 days [[1], [2], [3], [4], [5], [6], [7], [8], [9], [10], [11], [12], [13], [14], [15], [16], [17], [18], [19], [20], [21]] while the average time was 11 days. The median and average time between the last RT session and the next T-DXd administration was 11.0 days [[3], [4], [5], [6], [7], [8], [9], [10], [11], [12], [13], [14], [15], [16], [17], [18], [19], [20], [21], [22], [23], [24], [25]].

Patient and MBC characteristics and therapy are presented in Table 1.

**Table 1:** Patient, breast cancer and radiation therapy characteristics.

Characteristics	Patients (N = 54)
Gender	
Female	53 (98.15 %)
Male	1 (1.85 %)
Average age, (range)	61 years (39.6–85.7)
<65 years	38 (70.4 %)
>65 years	16 (29.6 %)
Type of MBC	
HER2+/HR+	14 (25.93 %)
HER2+/HR-	8 (14.81 %)
HER2 low/HR+	22 (40.74 %)
HER2 low/HR-	10 (18.52 %)
Dose of T-DXd	
4.4 mg/kg	4 (7.4 %)
5.4 mg/kg	50 (92.6 %)
Intent of RT	
Palliative	39 (72.2 %)
Curative	15 (27.8 %)
RT technique	
SRT	20 (37 %)
3D CRT	31 (57.4 %)
IMRT/VMAT	3 (5.6 %)
Indications	
Palliative bone irradiation	25 (46.3 %)
Intracranial SRS	14 (25.9 %)
WBRT	8 (14.8 %)
Extracranial SRT	4 (7.4 %)
Breast RT	3 (5.6 %)
RT Schedules	
8Gy in a single fraction	14 (25.93 %)
20Gy in 5 fractions	12 (22.22 %)
30Gy in 10 fractions	11 (20.37 %)
21Gy in 3 fractions	5 (9.26 %)
27Gy in 3 fractions	3 (5.56 %)
Other	9 (16.66 %)

MBC: Metastatic Breast Cancer; HER2: Human Epidermal Growth factor receptor 2.

HR: Hormone Receptor; T-DXd: Trastuzumab Deruxtecan.

RT: Radiotherapy; SRT: Stereotactic Radiotherapy.

SRS: Stereotactic Radio Surgery; WBRT: Whole Brain Radiotherapy; Gy: Gray.

**Table 2:** Line of administration according to the type of MBC.

Type of MBC	Line of T-DXd administration
HER2+ (22 patients)	2nd (18.2 %)
	3rd (31.8 %)
	4th (22.7 %)
	5th (18.2 %)
	6th (4.5 %)
	7th (4.5 %)
HER2 low/HR+ (22 patients)	2nd line (9.1 %)
	3rd line (22.7 %)
	4th line (18.2 %)
	5th line (27.3 %)
	6th line (9.1 %)
	7th line (4.5 %)
	9th line (4.5 %)
	11th line (4.5 %)
HER2 low/HR- (10 patients)	2nd line (20 %)
	3rd line (20 %)
	4th line (40 %)
	7th line (20 %)

### 3.2 Radiation therapy delivery

Among the 54 patients, 72.2 % received RT for palliative indications and 27.8 % for ablative RT, in the context of oligoprogression disease. Regarding irradiation techniques, 37 % of patients had stereotactic radiotherapy (SRT), 57.4 % 3D-conformal radiotherapy (3D CRT) and 5.6 % IMRT/VMAT. Indications consisted of palliative bone irradiation (46.3 %), or intracranial stereotactic radiosurgery (SRS) (25.9 %), whole brain radiotherapy (WBRT) (14.8 %), extracranial SRT (7.4 %) and breast RT (5.6 %). The fractionation could vary according to the indication between 1 fraction and 20 fractions.

Regarding the sites of palliative irradiation, we recorded 7 irradiations to the thoracic spine, 4 to the lumbar spine, 3 to the cervical spine, 2 to the acetabulum, 3 to the hip, 1 to the humerus, 1 to the iliac wing, 1 to the choroid, 1 to the cranial vault, and 1 to a cervical nodule.

Among the 3 breast irradiations (2 exclusive breast irradiations and one locoregional breast irradiation), the isodose corresponding to 50 % of the prescribed dose included a pulmonary volume ranging from 57 cc to 328 cc. Moreover, there were 8 palliative thoracic irradiations, involving the lungs.

The most commonly used fractionation scheme was 8 Gy (25.93 %) in a single fraction. Next was 20 Gy in 5 fractions (22.22 %), then 30 Gy in 10 fractions (20.37 %), 21 Gy in 3 fractions (9.26 %), 27 Gy in 3 fractions (5.56 %). Some stereotactic or palliative irradiations delivered in a single fraction were performed on the same day or the day after T-DXd administration, and the subsequent cycle was maintained as scheduled. There was no reduction in the dose of T-DXd during the radiation treatment or in the following cycles.

The data related to radiotherapy are presented in Table 3.

**Table 3:** Efficacy and toxicity results.

	Patients (N = 54)
Efficacy	
Complete response	12 (22.2 %)
Partial response	40 (74.1 %)
Stable disease	2 (3.7 %)
Radionecrosis	
Grade 1	3 (5.5 %)
Grade 2-4	0
Vomiting	
Grade 1	0
Grade 2	1 (1.8 %)
Grade 3-4	0
Diarrhea	
Grade 1	1 (1.8 %)
Grade 2-4	0
Interstitial lung disease	
Grade 1	3 (5.5 %)
Grade 2-4	0
Fatigue	
Grade 1	26 (48.1 %)
Grade 2	2 (3.7 %)
Grade 3-4	0
Radiation dermatis	
Grade 1	1 (1.8 %)
Grade 2-4	0

### 3.3 Safety and efficacy results

Grade 1 or 2 asthenia was observed in 51.8 % of patients, while only 16.6 % experienced other grade 1 or 2 adverse effects. Asthenia is a very common adverse effect. Grade 1 asthenia was observed in nearly 48.1 % of patients, while 3.7 % (n=2 cases) of patients experienced grade 2 asthenia. Aside from asthenia, the most common adverse effects were grade 1 radionecrosis (n=3 cases/5.5 %), grade 1 interstitial lung disease (n=3 cases/5.5 %), followed by grade 2 vomiting (n=1 case/1.8 %), grade 1 diarrhea (n=1 case/1.8 %), and grade 1 radiodermatitis (n=1 case/1.8 %). No patient had > grade 3 toxicity whatever the treated volume or fractionation. SRT patients had no grade >3 toxicity. All of these adverse effects were observed either during the post-radiation consultation or at the 3-month follow-up but had resolved spontaneously. No late toxicities were recorded. Three cases of grade 1 radionecrosis were observed among the 14 patients treated with brain SRS and 8 with WBRT.

Additionally, the three cases of grade 1 radionecrosis occurred after SRS. Regarding the three cases of grade 1 interstitial lung disease, they occurred in the context of breast irradiation and two palliative thoracic spine irradiations. The grade 2 vomiting episode occurred after total brain irradiation. Finally, the diarrhea episode occurred following irradiation of the iliac wing.

Regarding potential associated treatments, to prevent potential intracranial hypertension, corticosteroid therapy was initiated in seven patients. The other patients had no additional treatment besides radiotherapy and T-DXd.

Furthermore, there were no cases of permanent discontinuation of T-DXd therapy related to RT. We observed a temporary interruption of T-DXd in 5.6 % (n=3 cases) of patients.

After a first median assessment of six months, 22.2 % of patients had a complete response, 74.1 % experienced a partial response, and 3.7 % had a stable disease.

The data related to efficacy and toxicity are presented in Table 3.

## 4 Discussion

The efficacy of T-DXd in inoperable or metastatic HER2-positive breast cancer, following progression during T-DM1 therapy, has been established. This was confirmed in the randomized Phase III trials, DESTINY-Breast 02 and DESTINY-Breast 03, involving patients with inoperable or metastatic HER2-positive breast cancer who had previously received trastuzumab- and taxane-based therapies [6]. DESTINY-Breast 04 is the first effective anti-HER2 targeted therapy option for patients with HER2-low breast cancer [13]. The efficacy of T-DXd in patients with HER2-positive/low breast cancer is currently being evaluated in several clinical trials, such as DESTINY-Breast 05 through DESTINY-Breast 12 [14]. Recently presented at ASCO 2024, DESTINY Breast 06 establishes T-DXd as a standard of care following at least one endocrine-based therapy for patients with HER2-low and HER2-ultralow, HR + MBC [15]. These trials directly challenge the current standard treatment protocols at various stages of therapy, with the hope that other Phase III studies will further revolutionize clinical practice. DESTINY-Breast 05 is a phase 3, multicenter, randomized, open-label, active-controlled trial comparing T-DXd to T-DM1 in patients with high-risk HER2-positive primary breast cancer who have residual invasive disease in the breast or axillary lymph nodes following neoadjuvant therapy. DESTINY-Breast 05 offers clinicians the choice to perform breast irradiation concurrently with T-DXd or to start T-DXd after irradiation [15]. The conclusions drawn from these ongoing clinical trials are expected to play a pivotal role in shaping the future treatment paradigm for HER 2 positive and HER2 low breast cancer [16].

Regarding the specific potential side effects of T-DXd, interstitial lung disease is a particular adverse reaction associated with T-DXd that requires monitoring patients' respiratory signs and symptoms and early intervention to prevent severe consequences [17]. Another specific adverse reaction linked to T-DXd is left ventricular dysfunction, identified as a warning and caution in the United States Prescribing Information. T-DXd treatment hasn't been studied in patients with a significant history of cardiac disease or left ventricular ejection fraction <50 %. However, given that LVEF is a recognized adverse event with HER2-targeted therapies, it should be emphasized as a warning [18]. Additional adverse events associated with T-DXd include nausea, fatigue, vomiting, and alopecia occurring at different frequencies, primarily attributed to specific payloads [19,20].

Limited data exists in the literature regarding the combination of RT and T-DXd. Table 4 summarizes the current available data. Regarding the risk of CNS toxicity, the DEBBRAH phase II study demonstrated that the potential intracranial activity of T-DXd and stated that radiation is feasible with manageable toxicity in patients with HER2-positive and HER2-low breast cancer who predominantly received WBRT and/or SRS. However, the authors did not specify the timing between irradiation and sequential administration of T-DXd [20,21]. These findings align with our TENDANCE study results that showed only limited grade 1 adverse events following brain irradiation.

**Table 4:** Studies regarding the combination of Radiotherapy and Trastuzumab Deruxtecan.

	Design	Number of patients	Intervention	Administration	Toxicity
DEBBRAH Trial [21]	Phase II	21	T-DXD ± WBRT or SRS	NR	Limited data Feasible, without additional toxicity
Visani et al. (Poster Breast ESMO, 2024) [22]	Retrospective Study	16	T-DXd + RT	Concomitant	Fatigue G3 (n = 1); Interstitial lung disease G2 (n = 1); No radionecrosis
Bouziane et al. [23]	Retrospective Study	33	T-DXd + RT	Concomitant	Grade 2 (asthenia, mucositis, cardiac decompensation, and diarrhea) (n = 7); Late toxicities (n = 7)
TENDANCE Study (Debbi K. et al.)	Retrospective Study	54	T-DXd + RT	Concomitant	Radionecrosis grade 1 (n = 3); vomiting grade 2 (n = 1); diarrhea grade 1 (n = 1); radiation dermatis grade 1 (n = 1)

Regarding interstitial pneumonitis after T-DXd administration, the rate in Destiny-Breast03 study was 15 % [14]. The additional risk with concurrent RT is questioned. In our study, 1 and 2 patients underwent respectively whole breast irradiation with or without regional nodal areas. Despite the partial involvement of the lung (328 cc and 57 cc), no patient exhibited pulmonary symptoms. In our cohort, with a median follow-up of 9 months, 3 cases of grade 1 interstitial lung disease.

In one retrospective study presented recently at ESMO Breast Cancer meeting, Visani et al. reported among 52 patients treated with T-DXd, 16 patients who received RT either immediately before (within a month) or during T-DXd with 17 RT treatments (one patient was treated with RT twice) [22]. The median total RT dose prescription was 30 Gy (range 8–48), with a median number of fractions of 3 (range 1–15). The most frequently treated site was bone (47.1 % of cases; N = 8/17), followed by the brain (29.4 %; N = 5/17). Among the patients who received RT concurrently with T-DXd, the toxicity was moderate. Overall, only one case of fatigue grade 3 was observed. Interstitial lung disease grade 2, that led to T-DXd discontinuation was observed in 1 case. No radionecrosis events was observed among the 4 patients treated with intracranial RT. The favorable tolerance of the combination of RT and T-DXd, with a limited number of events and low grades, is consistent with the results of our study. The only difference is that we did not observe any cases of grade 2 interstitial lung disease.

Recently, a retrospective study published by Bouziane presented the results of this association. Among the 33 patients included, 7 experienced acute adverse effects, and 7 had late toxicity [23].

The question of suspending T-DXd just before RT start is discussed for routine practice. The elimination half-life of T-DXd is approximately 7 days [24]. The general cautionary principle recommends suspending treatment for a duration equivalent to 5 half-lives, which is 35 days. In clinical practice, a strictly sequential treatment is difficult to apply. In our study, the average time between the last T-DXd infusion and the first RT session was 10 days, with very limited toxicities. Thus, concurrent irradiation with the administration of T-DXd seems to be feasible.

For the other ADCs, clear recommendations exist concerning trastuzumab emtansine (TDM-1) and concurrent RT. T-DM1 and concomitant RT can be realized during adjuvant locoregional RT for breast cancer, although this may increase pulmonary and/or cutaneous toxicity. On the other hand, T-DM1 and concomitant WBRT and ablative intracranial SRS must not be offered, due to increased risk of radionecrosis [20,25]. T-DXd demonstrates excellent intracranial efficacy, superior to that of T-DM1 [6], and very few symptomatic radionecrosis seem to be observed when SRS is performed [22,26,27].

Unfortunately, data on sacituzumab govitecan are lacking in the literature.

This study has several limitations. The small sample size and retrospective design limit the generalizability of the findings. The lack of baseline adverse event data makes it difficult to assess safety accurately. The heterogeneity of tumor sites and treatment techniques may have influenced the results. Finally, the short follow-up period restricts the ability to evaluate long-term outcomes.

## 5 Conclusion

To best our knowledge, TENDANCE cohort is to date the largest study evaluating safety and efficacy of concurrent RT in patients treated with T-DXd. These findings indicate minimal and manageable grade 1–2 toxicity, with an optimal clinical benefit observed from a non-discontinued combination of T-DXd and RT. Our results on concurrent T-DXd and RT are encouraging in terms of safety and efficacy. However, these findings require confirmation through longer-term follow-up and additional prospective studies.

## Support

No support was received for this project.

## CRediT authorship contribution statement

**K. Debbi:** Writing – review & editing, Writing – original draft, Visualization, Validation, Resources, Methodology, Investigation, Formal analysis, Data curation, Conceptualization. **M.A. Benderra:** Writing – review & editing, Visualization, Investigation, Data curation. **J. Medioni:** Writing – review & editing, Visualization, Investigation, Data curation. **C. Durdux:** Writing – review & editing, Validation, Funding acquisition. **N.H. To:** Writing – review & editing, Methodology. **C. Boukhobza:** Writing – review & editing, Visualization. **N. Grellier:** Visualization, Data curation. **A. Benmaziane:** Visualization, Validation, Data curation. **L. Monnier:** Validation. **J. Gligorov:** Validation, Supervision, Methodology, Data curation. **E. Assaf:** Visualization, Validation, Methodology, Data curation. **Y. Belkacemi:** Writing – review & editing, Writing – original draft, Visualization, Validation, Supervision, Resources, Project administration, Methodology, Investigation, Formal analysis, Data curation, Conceptualization.

## Declaration of competing interest

None.
